# Neutrophilic cholangitis associated with intracholangiocytic *Hammondia* protozoa confirmed in 2 dogs and suspected in 2 dogs

**DOI:** 10.1177/03009858261449082

**Published:** 2026-05-11

**Authors:** Eunju (April) Choi, Devinn M. Sinnott, Amira A. Ahmed, Anibal G. Armien, Karen Shapiro, Manigandan L. Virapin

**Affiliations:** 1 University of California, Davis, Davis, CA; 2 Louisiana State University, Baton Rouge, LA; 3 Assiut University, Asyut, Egypt; 4 Cornell University, Ithaca, NY

**Keywords:** canine, cholangitis, cholecystitis, gallbladder, *Hammondia*, liver, sarcocystidae

## Abstract

Four dogs with neutrophilic cholangitis were identified with intracholangiocytic apicomplexan protozoa. Two cases were biopsies, and both cases had gallbladder involvement. The other two cases were unexpected findings on autopsy. Immunohistochemistry against *Toxoplasma gondii* on all cases labeled the zoites. *Internal transcribed spacer-1* polymerase chain reaction (PCR) performed on formalin-fixed paraffin-embedded samples was successful in the 2 biopsy cases, and subsequent sequencing identified >99% sequence identity to *Hammondia* sp. Of the biopsied dogs, 1 dog recovered successfully after antiprotozoal treatment while the other dog was euthanized due to poor response. The 2 autopsy cases were given immunosuppressive medication. *Hammondia* are within the Sarcocystidae family of apicomplexan parasites and are closely related to *Neospora caninum*. This study and the current literature support that *Hammondia*, specifically *Hammondia heydorni*, an organism that was previously considered nonpathogenic or minimally pathogenic, can rarely exhibit tropism to the biliary system and cause neutrophilic cholangitis, cholangiohepatitis, and cholecystitis in dogs.

Cholangitis/cholangiohepatitis refers to inflammation of the intrahepatic biliary system.^5^ Cholangitis typically refers to the inflammation centered on the bile ducts without expansion into the hepatic parenchyma while cholangiohepatitis can be used when the bile duct or ductule-centered inflammation is severe enough to breach the limiting plate, damaging the hepatocytes.^5^ Neutrophilic cholangitis/cholangiohepatitis in dogs has been mostly considered to be caused by ascending bacterial infections from the gastrointestinal tract.^14,15,21^ Recently, an aseptic pathogenesis has been proposed based on frequently negative culture results^5,21^; however, these dogs have been given antibiotics, which is suspected to have affected the culture results.

Neutrophilic cholangitis has been documented with intrabiliary coccidiosis in a dog.^10^ While a range of coccidian species were suspected, the etiologic agent was not determined.^10^ Sarcocystidae-like zoites were identified cytologically in canine bile with neutrophilic inflammation, and PCR of bile revealed a partial *18S rDNA* sequence with 100% identity to *Hammondia heydorni* and *Hammondia triffittae*, with the patient surviving after treatment with clindamycin, enrofloxacin, and denamarin.^6^ *H. heydorni* was identified as a cause of acute cholangiohepatitis in a dog fed a raw food diet based on biochemical findings, but histology was not performed.^2^ These cases suggest that hammondiosis can be associated with biliary inflammation in dogs.

*H. heydorni* and *H. triffittae* are members of the genus *Hammondia* along with *Hammondia hammondi* and *Hammondia pardalis.*^13^ *H. heydorni* and *H. triffittae* were identified to be monophyletic with *Neospora caninum*, and the other two *Hammondia* species are monophyletic with *Toxoplasma gondii*.^13^ While the definitive host for *H. hammondi* and *H. pardalis* are felids, the definitive host for *H. heydorni* are canids, both domestic and wild. The definitive host for *H. triffittae* is the red fox and Arctic fox.^1^ Of note, *H. triffittae* has not been identified in the domestic dog.

The prevalence of *H. heydorni* in dogs is not well understood. In a study that screened for fecal *H. heydorni* DNA in wild canids in and around an Ohio wildlife conservation center, 3 of 51 samples with coccidian DNA were positive for *H. heydorni.*^12^ In routine fecal parasitologic examination, the distinction between *N. caninum* and *H. heydorni* is not possible based on the morphology of oocysts alone, and hence, without genetic testing, the actual agent cannot be confirmed.

*Hammondia* species have mostly been considered nonpathogenic because most oocysts are found in asymptomatic dogs^20^; however, there have been reports of identifying these oocysts in dogs with diarrhea and rarely anorexia,^1,6,18^ suggesting that some infected dogs may be clinical. Histopathologic lesions associated with this coccidian parasite in clinical cases have not been documented.

This brief report summarizes the clinical, microscopic, and immunohistochemical features of 4 dogs with protozoal cholangitis of which 2 were confirmed by PCR to be hammondiosis, and 2 were PCR negative but strongly suspected to be *Hammondia* sp. based on similar biliary tissue tropism.

Signalment, geographic location, and clinicopathologic findings are summarized in Table 1. Case 1 was a biopsy submission from 2018 of the liver and gallbladder from a 10-year-old, female spayed, Bichon Frise from Philadelphia that presented to an emergency clinic with vomiting, anorexia, fever, and elevated liver enzymes. Cholecystectomy and liver biopsy were performed, and samples were submitted in 10% neutral-buffered formalin to the Cornell University Animal Health Diagnostic Center (AHDC).

**Table 1.** Signalment, geographic location, clinical signs, selected liver enzyme values, and immune status.

	Case 1	Case 2	Case 3	Case 4
Age (y)	11	5	13 (suspected)	9
Sex	FS	FS	MC	M
Breed	Bichon Frise	Miniature pinscher	German shorthair pointer	Boxer
Residence	Pennsylvania	Florida	Northern California	Northern California
Clinical signs	Vomiting, anorexia, fever for 1 month.	Progressive vomiting for 2 weeks.	Rescued 4.5 months prior and diagnosed with IBD. Euthanized due to progressive weight loss and anorexia.	Received chemotherapy for multicentric lymphoma, then euthanized due to progression.
Biochemistry				
ALT (IU/L)	812	219	NA	131
ALP (IU/L)	1586	3355	NA	832
GGT (IU/L)	22	24	NA	NA
T. bili (mg/dL)	1.8	NA	NA	0.5
Immunosuppression	Unknown	Unknown	Yes	Yes
Submission	AHDC; biopsy	AHDC; biopsy	UC Davis; autopsy	UC Davis; autopsy

Abbreviations: Y, year; FS, female spayed; MC, male castrated; M, male; IBD, inflammatory bowel disease; ALT, alanine aminotransferase; NA, not available; ALP, alkaline phosphatase; GGT, gamma glutamyl transferase; T. bili, total bilirubin; AHDC, Animal Health Diagnostic Center; UC Davis, University of California, Davis.

Case 2 was a biopsy submission from 2015 of liver and gallbladder from a 4-year-old, female spayed, miniature pinscher from Florida that presented with a 2-week history of vomiting and increased liver enzymes. Cholecystectomy and liver biopsy were performed, and samples were submitted in 10% neutral-buffered formalin to the Cornell University AHDC.

Routine processing and paraffin embedding to yield 4-μm-thick sections that were stained with hematoxylin and eosin for microscopic examination were performed by the AHDC Histology Laboratory.

Case 3 was an autopsy case from 2015 of an approximately 13-year-old, male castrated, German shorthaired pointer that was rescued in California 4.5 months prior to euthanasia. The patient was diagnosed with severe inflammatory bowel disease within 2 months after the rescue at the University of California, Davis (UC Davis), Veterinary Medical Teaching Hospital. The inflammatory bowel disease was not controlled, and the patient was given 2 doses of dexamethasone intravenously and then was managed with prednisone and cyclosporine for a month until euthanasia. When this treatment was initiated, fecal flotation was performed, which identified *Neospora*-like oocysts that were 10–13 μm in diameter and were noted to be difficult to morphologically distinguish from *H. heydorni*. In the final month, the patient had severe side effects from the prednisone therapy and was euthanized due to poor quality of life. The dog was submitted for autopsy to the UC Davis Anatomic Pathology Service.

Case 4 was a 9-year-old, male intact, boxer who resided in California and was treated at UC Davis in 1991 for grade IIIA lymphoma for 1 month. The lymphoma quickly progressed, and the patient was euthanized due to poor quality of life and submitted for autopsy to the UC Davis Anatomic Pathology Service.

A routine autopsy was performed for cases 3 and 4, and major organ systems, apart from the central nervous system, were examined grossly and histologically. For both cases 3 and 4, the gallbladder was not mentioned in the gross report and was unavailable for histologic assessment.

Histologic examination of the biliary system revealed intracholangiocytic apicomplexan protozoal organisms associated with neutrophilic cholangitis/cholangiohepatitis in all 4 cases, and proliferative cholecystitis in cases 1 and 2 that had gallbladder available for examination. Specifically, the liver of case 1 had large numbers of neutrophils expanding the portal stroma and obscuring the basement membrane of bile ducts (Fig. 1a). Occasional bile ducts were filled with a small number of neutrophils. In occasional portal tracts, where the portal vein was expected, there was a mat of fibrin supportive of portal thrombosis. Mostly in the apical cytoplasm of cholangiocytes were approximately 10-μm-diameter, round, variably basophilic structures surrounded by a 1- to 2-μm clear space interpreted as schizonts. Occasionally, a few 2 × 5-μm crescent-shaped zoites formed a radiating or palisading pattern (Fig. 1a, inset). In addition to the cholangitis, a random hepatitis was present, characterized by occasional random aggregates of hepatocytes disrupted by neutrophils and fibrin. The mucosa of the gallbladder of case 1 was thickened due to a marked proliferation of epithelial cells forming papillary fronds, and the lamina propria was infiltrated with mostly lymphocytes and plasma cells with small numbers of superficial neutrophils. Case 2 had moderate luminal neutrophilic cholangitis and neutrophilic involvement of the portal stroma. Bile ducts were often mildly to moderately dilated. Ducts with intracholangiocytic protozoa were difficult to find, but when present, there were multiple zoites in the affected duct. The gallbladder of case 2 was similarly affected by chronic proliferative and lymphoplasmacytic cholecystitis (Fig. 1b) as described in case 1 with a few superficial epithelial protozoa (Fig. 1b, inset). The liver of case 3 had neutrophilic cholangitis, similar to case 2, but in addition, the cholangiocytes were attenuated, and the surrounding portal stroma was expanded by fibrosis and infiltrated with neutrophils, histiocytes, fibroblasts, and fewer lymphocytes and plasma cells (Fig. 1c). Similar to case 2, rare bile ducts had intracholangiocytic protozoal schizonts (Fig. 1c, inset). Neoplastic lymphocytes dominated the liver of case 4, expanding portal and central vein adventitia, as well as sinusoids; however, rare bile ducts had a subtle neutrophilic cholangitis, and one duct in particular had a dozen, 3- to 5-µm-diameter meronts (Fig. 1d).

**Figure 1.**
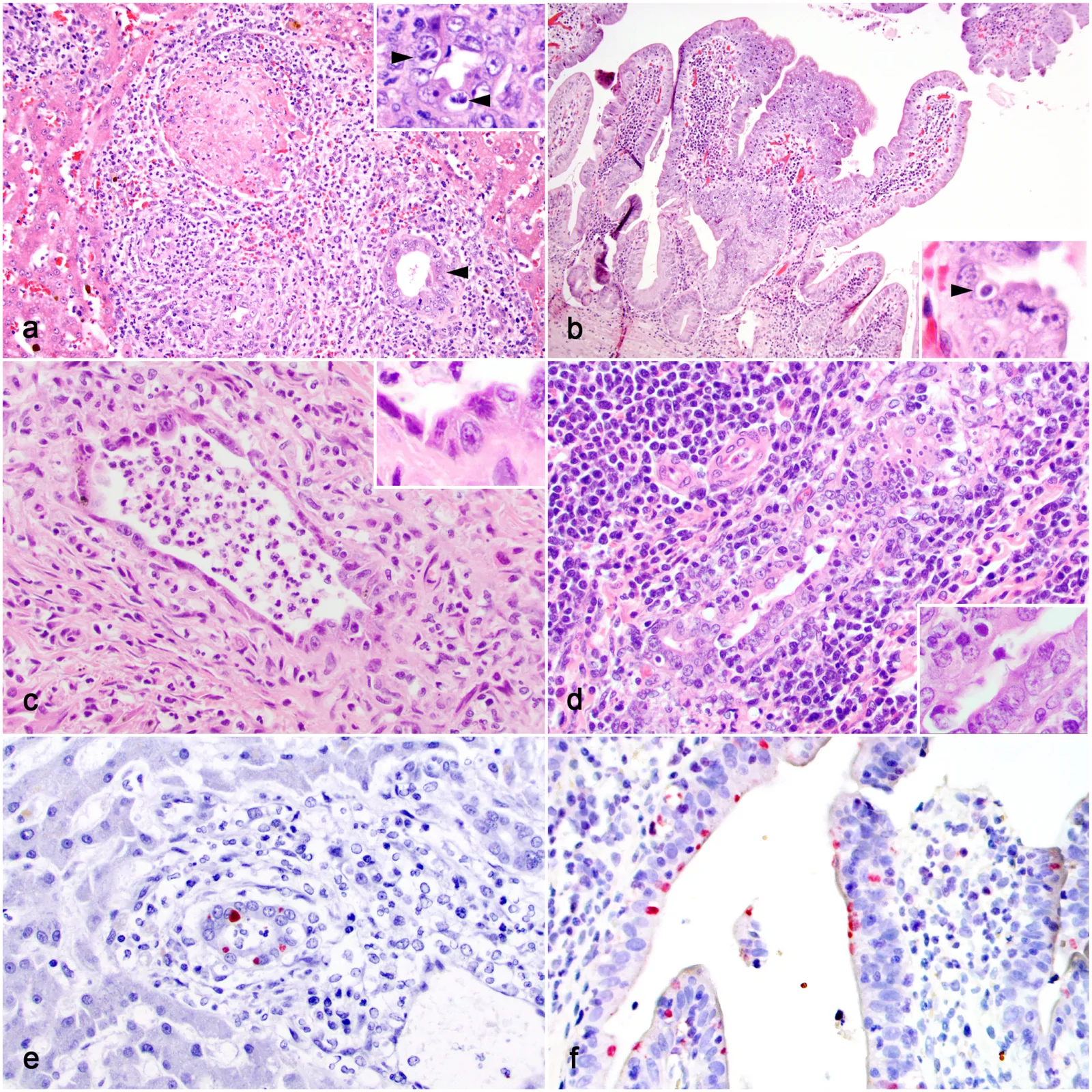
Histopathologic features of canine cholangitis and cholecystitis with apicomplexan protozoal infection. (a) Liver. The portal tract is expanded by neutrophils. The bile duct is mildly ectatic, and the nuclei of cholangiocytes are disorganized (arrowhead). An acute fibrin thrombus obliterates the portal vein. A bile duct with intracytoplasmic protozoal organisms suspected to be undergoing schizogony in parasitophorous vacuoles are highlighted in the inset (arrowhead). Case 1. Hematoxylin and eosin (HE). (b) Gallbladder. The mucosa is hyperplastic forming anastomosing fronds, and the cholangiocytes and lamina propria are infiltrated by moderate numbers of inflammatory cells. Inset: The superficial epithelium is jumbled, and there is a suspected schizont or meront (arrowhead) and a mitotic figure. Case 2. HE. (c) Liver. The bile duct epithelium is attenuated, and the lumen is ectatic and filled with neutrophils. The portal stroma is expanded by collagen and infiltrated by mixed inflammatory cells. Inset: Protozoa undergoing schizogony. Case 3. HE. (d) Liver. The portal tract is significantly infiltrated by neoplastic lymphocytes, but the bile duct is surrounded by a small number of neutrophils and infiltrated with abundant, 3- to 5-μm-diameter, round meronts in small vacuoles. The inset highlights schizonts. Case 4. HE. (e) Liver. Immunohistochemistry (IHC) for *Toxoplasma gondii* highlights the intracholangiocytic organisms of various sizes. Case 2. (f) Gallbladder. Florid, predominantly apical, intracytoplasmic immunopositive protozoa are identified with *T. gondii* IHC. Case 1.

Immunohistochemistry (IHC) for *T. gondii* and *N. caninum* was performed at the AHDC on the liver of all 4 cases and gallbladder of cases 1 and 2 (methods detailed in the Supplemental Materials).

The IHC for *T. gondii* highlighted the intracholangiocytic organisms in the liver of all 4 cases (Fig. 1e), as well as the superficial epithelium of the gallbladder of cases 1 (Fig. 1f) and 2. The organisms were florid in the biliary epithelium of the liver and gallbladder in case 1 and were also identified free in the lumen of disrupted intrahepatic bile ducts. Compared to case 1, IHC only identified a few bile ducts with intraepithelial protozoa in cases 2–4. IHC highlighted smaller protozoal elements not easily appreciated with hematoxylin and eosin alone. Similarly, in the gallbladder of case 2, there were regions where the IHC highlighted a few superficial epithelial cytoplasmic protozoa. The protozoa were not identified in hepatocytes of any of the cases or in the regions of fibrin thrombosis or random hepatitis in case 1. In the gallbladder, the organisms were not identified in the lamina propria or the mucosal glands. IHC for *Neospora* spp. did not highlight the organisms.

Formalin-fixed, paraffin-embedded gallbladder tissue from case 1 was ultrastructurally examined (methods detailed in the Supplemental Materials). Electron microscopy confirmed intracytoplasmic eukaryotic organisms of up to 1.08 × 1.86 μm to be apicomplexan protozoa by the presence of rhoptry, micronemes, rough endoplasmic reticulum, and a mitochondrion (Supplemental Figure S1); however, the quality of images did not allow further characterization of the fine structure.

Molecular diagnostics were attempted on all 4 cases on formalin-fixed paraffin-embedded scrolls of the liver (cases 1, 3, and 4) or gallbladder (case 2; methods detailed in the Supplemental Materials). Amplification of the *ITS-1* locus yielded a band for cases 1 and 2, the sequences of which showed greatest identity (>99%) to *Hammondia sp.* Amplification was unsuccessful for cases 3 and 4 despite testing multiple replicates for the available specimens.

Biliary tissue tropism has not been reported for protozoan species belonging to the family Sarcocystidae in veterinary species until recently and led to the molecular identification of *Hammondia* species in these cases.^2,6^

*T. gondii* and *N. caninum*, two major pathogenic apicomplexan protozoa of the dog, infect hepatocytes but not cholangiocytes. Biliary coccidiosis is reported in other veterinary species. In the neighboring family Eimeriadae, there are two primary biliary coccidia, *Eimeria stiedae* of the rabbit and *Eimeria furonis* of ferrets, that cause proliferative cholangitis.^22^ *Cryptosporidium parvum*, a member of the suborder Eimeriorina that encompasses families Sarcocystidae and Eimeridae, can also infect cholangiocytes in addition to enterocytes in immunocompromised primates.^8^

*H. heydorni* has mostly been regarded as a nonpathogenic organism as experimental studies have not produced clinical signs despite infected dogs shedding oocyts.^11^ Some studies have documented mild diarrhea in normal and immunocompromised dogs.^1,3,17^ Case 3 had coccidia identified by fecal exam that were presumed nonpathogenic, and immunosuppressive therapy for inflammatory bowel disease was initiated, which may have exacerbated the cholangitis. It is difficult to speculate how and under what conditions *H. heydorni* infects the canine biliary tree. It is unclear why and how *H. heydorni* causes a neutrophilic cholangitis instead of a more necrotizing process, as expected with other hepatic protozoal infections, such as those with *T. gondii* or *N. caninum*. A plausible speculation may be associated with the innate immunity of biliary epithelium^4^ and the fact that damaged cholangiocytes express interleukin-8, a neutrophilic chemotactant.^7^

Identifying cases of biliary coccidiosis in dogs was difficult. First, the organisms were generally scant, and identifying zoites within degenerate and inflamed bile ducts was challenging. Second, the 3 cases identified following the initial case were considered “atypical” *Toxoplasma* infections given the strong positive IHC labeling but odd tissue tropism. Cross-immunoreactivity within the family Sarcocystidae with the commercial *Toxoplasma* antibody has been previously reported^9,16,19^; hence, molecular identification is required to accurately determine etiology. PCR failed to positively identify the organism in 2 of 4 cases, both of which were necropsy cases with an unknown duration of formalin fixation. The 2 cases that successfully amplified parasite DNA with PCR were biopsy samples that generally have shorter fixation times and had more protozoa identified with IHC than the other two cases. Despite repeatedly negative PCR results for the 2 necropsy cases (3 and 4), the specific tissue tropism is strongly suggestive of *H. heydorni* based on our 2 positive results and the current literature.^2,6^ False-negative results may be attributed to insufficient sample, poor DNA quality associated with prolonged formalin-fixation time, and/or large amplicon size for formalin-fixed paraffin-embedded samples.

Given how sporadic this disease has been reported, we believe the disease prevalence is low. However, pathologists should bear in mind that neutrophilic cholangitis in a dog can be due to *Hammondia* sp., specifically *H. heydorni*, and should attempt to identify this organism on hematoxylin and eosin–stained sections or with *Toxoplasma* IHC if deemed warranted. Fecal testing to visualize oocysts can also be useful to corroborate infection.

## Supplemental Material

sj-pdf-1-vet-10.1177_03009858261449082 – Supplemental material for Neutrophilic cholangitis associated with intracholangiocytic Hammondia protozoa confirmed in 2 dogs and suspected in 2 dogs Supplemental material, sj-pdf-1-vet-10.1177_03009858261449082 for Neutrophilic cholangitis associated with intracholangiocytic Hammondia protozoa confirmed in 2 dogs and suspected in 2 dogs by Eunju (April) Choi, Devinn M. Sinnott, Amira A. Ahmed, Anibal G. Armien, Karen Shapiro and Manigandan L. Virapin in Veterinary Pathology
