# Supplementary material for: Neutrophilic cholangitis associated with intracholangiocytic Hammondia protozoa confirmed in 2 dogs and suspected in 2 dogs
Source: Vet Pathol. 2026 May 11;63(5):837–41. doi: 10.1177/03009858261449082 (PMC13534764; doi:10.1177/03009858261449082)
Supplement: Supplementary file 1 [file sj-pdf-1-vet-10.1177_03009858261449082.pdf]

**Supplemental Material**

**Neutrophilic cholangitis associated with intracholangiocytic *Hammondia* protozoa confirmed in 2 dogs and suspected in 2 dogs**

Eunju (April) Choi, Devinn M. Sinnott, Amira A. Ahmed, Anibal G. Armien, Karen Shapiro, Manigandan L. Virapin

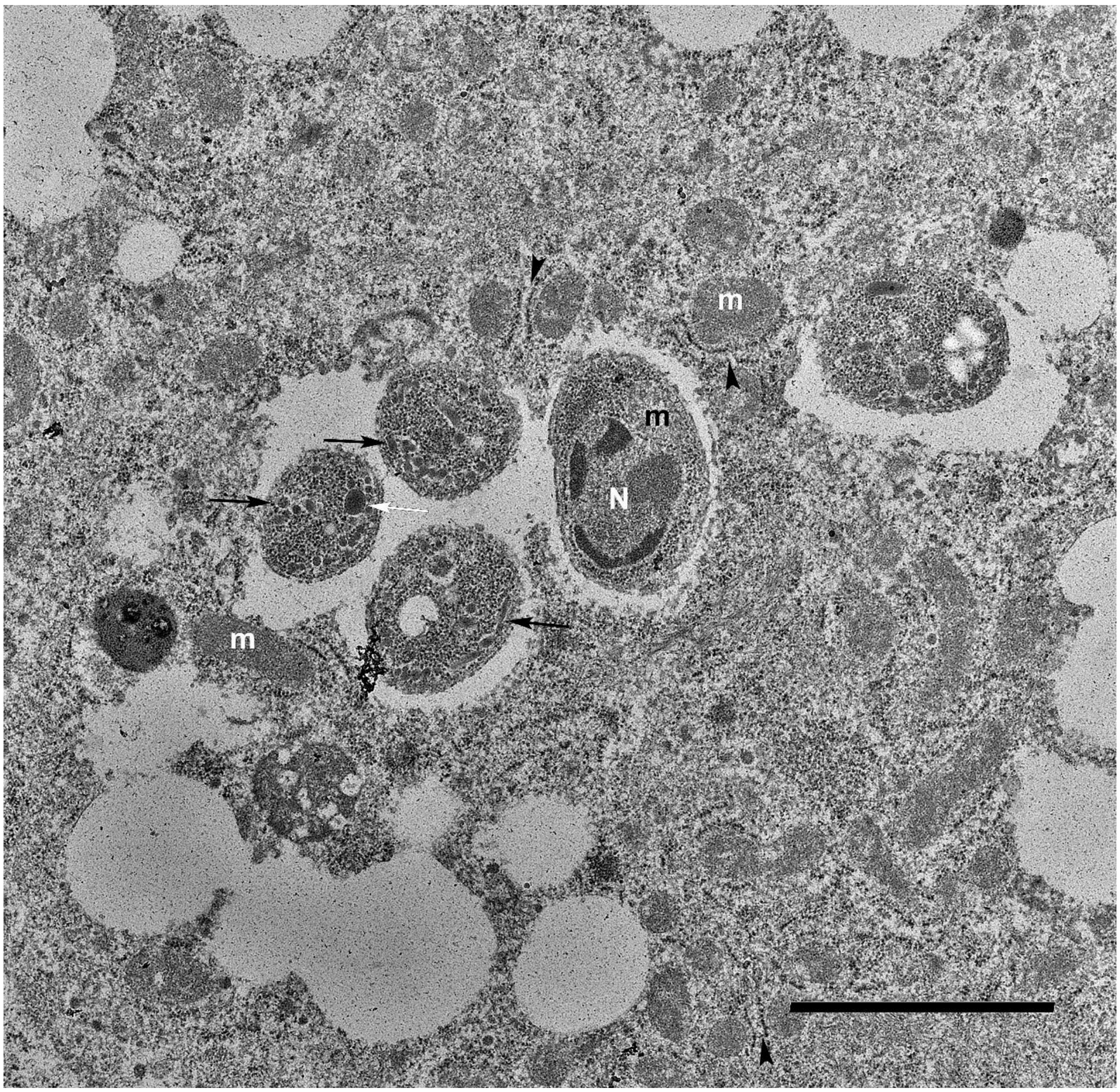


**Supplemental Figure S1**. Electron microscopy of a gallbladder epithelial cell from case 1 depicting intracytoplasmic eukaryotic parasites within parasitophorous vacuoles. Eukaryotic parasites display one or more of the following structures: nucleus (N), microneme (black arrow), rhoptry (white arrow), and mitochondria (black m) consistent with apicomplexan protozoa. The gallbladder epithelial cell contains mitochondria (white m) and rough endoplasmic reticulum (black arrowhead). Lead citrate-uranyl acetate resin-embedded section obtained from formalin-fixed paraffin-embedded tissue.

## Supplemental Methods

*Toxoplasma gondii and Neospora caninum Immunohistochemistry*

Mouse polyclonal antibody against *Toxoplasma gondii* (US Biological, catalog #T8076-10) and mouse monoclonal antibody against *Neospora caninum* (US Biological, catalog #369242) were used with the Leica BOND Max automated IHC staining system. Briefly, slides were dewaxed, underwent heat epitope retrieval for 30 minutes, and the primary antibody diluted at 1:2000 was applied to the slide for 60 min. This was followed by application of the secondary antibody for 30 min and red detection kit polymer for 15 min. The slide was then stained with hematoxylin for 5 min. Positive controls were liver sections of confirmed respective infections. Negative controls were produced by incubation with primary antibody diluent.

*Electron Microscopy*

Formalin-fixed paraffin embedded tissues were gently deparaffinized and rehydrated in a decreasing alcohol gradient. Tissues were prepared by methods previously described.[1] Briefly, tissues were trimmed to 1 to 2 cubic mm, post-fixed in Karnovsky solution. Tissue was processed, and resin-embedded in an automated tissue processor. Ultrathin sections were obtained and stained with uranyl acetate and lead citrate.

*Molecular diagnostics*

Genomic DNA was extracted from three 10-μm scrolls of formalin-fixed, paraffin-embedded liver tissue from all cases, and from gallbladder tissue for case 2. Tissue sections were deparaffinized using a xylene substitute (VWR, 89370-090) followed by two 100% ethanol washes, and DNA was then extracted using a DNeasy Blood and Tissue Kit (Qiagen, Valencia, CA) following the manufacturer's protocol with one modification: 180 μl buffer ATL and 30 μl proteinase K were added to each sample and incubated at 56 °C overnight.

A 455 bp fragment of the *internal transcribed spacer 1* (*ITS-1*) region was amplified with a nested assay using external primers (ITS1DF: 5’ -TACCGATTGAGTGTTCCGGTG-3’ and ITS1DR: 5’ -GCAATTCACATTGCGTTTCGC-3’) and internal primers (ITS1diF: 5’ -CGTAACAAGGTTTCCGTAGG-3’ and ITS1diR: 5’ -TTCATCGTTGCGCGAGCCAAG-3’) as previously described.[2] Each 50 µL external reaction contained 22 µl nuclease-free water, 25 µl AmpliTaq Gold 360 master mix (Applied Biosystems, Foster City, CA), 0.5 µl of each external primer (50 µM), and 2 µl DNA. Thermal cycling conditions were as follows: initial denaturation at 94 °C for 3 min; 35 cycles of 95 °C for 40 sec, 58 °C for 40 sec, and 72 °C for 90 seconds; followed by a final extension at 72 °C for 4 min. Each 50 µL internal reaction contained 23 µl nuclease-free water, 25 µl AmpliTaq Gold 360 master mix, 0.5 µl of each internal primer (50 µM), and 1 µl external reaction product. Thermal cycling conditions were as follows: initial denaturation at 94 °C for 3 min; 35 cycles of 95 °C for 40 sec, 59 °C for 40 sec, and 72 °C for 90 seconds; followed by a final extension at 72 °C for 4 min. Negative controls (nuclease-free water) and a positive control (DNA extracted from a *Sarcocystis neurona* isolate cultured from the brain of a harbor seal) were included in each PCR run to confirm the absence of contamination and to confirm successful amplification of target DNA.

Amplicons were resolved by electrophoresis on 2% agarose gels stained with RedSafe (Bulldog Bio, Portsmouth, NH) and visualized using a Gel Doc EZ Imaging System (BioRad, Hercules, CA). Bands of expected size were purified using a *Qiaquick Gel Extraction Kit* (Qiagen, Valencia, CA) and submitted for Sanger sequencing at Genewiz (South San Francisco, CA) using the internal primer set. Consensus sequences were pairwise aligned and trimmed using Geneious (Dotmatics, Auckland, New Zealand). Consensus sequences were analyzed using the BLASTn algorithm against the NCBI Nucleotide Collection database (https://blast.ncbi.nlm.nih.gov).

Initial DNA extraction and amplification of the *ITS-1* locus did not yield bands for cases 3 and 4. An additional three replicates each consisting of two 20-µm formalin-fixed paraffin-embedded scrolls were processed as described above for cases 3 and 4. Amplification was not successful for any of these additional replicates for either case.

## References


1. Armién AG, Wolf TM, Mor SK, et al. Molecular and Biological Characterization of a Cervidpoxvirus Isolated From Moose with Necrotizing Dermatitis. *Vet Pathol*. 2020 Mar;57:296–310.
2. Rejmanek D, VanWormer E, Miller MA, Mazet JAK, Nichelason AE, Melli AC, Packham AE, Jessup DA, Conrad PA. Prevalence and risk factors associated with *Sarcocystis neurona* infections in opossums (*Didelphis virginiana*) from central California. *Vet Parasitol*. 2009;166:8-14.
